# Small airway fibroblasts from patients with chronic obstructive pulmonary disease exhibit cellular senescence

**DOI:** 10.1152/ajplung.00419.2022

**Published:** 2023-12-27

**Authors:** Catherine L. Wrench, Jonathan R. Baker, Sue Monkley, Peter S. Fenwick, Lynne Murray, Louise E. Donnelly, Peter J. Barnes

**Affiliations:** ^1^Airway Disease Section, National Heart and Lung Institute, Imperial College, London, United Kingdom; ^2^Bioscience COPD/IPF, Research and Early Development, Respiratory & Immunology (R&I), Biopharmaceuticals R&D, AstraZeneca, Cambridge, United Kingdom; ^3^Translation Science and Experimental Medicine, Research and Early Development, Respiratory & Immunology (R&I), Biopharmaceuticals R&D, AstraZeneca, Gothenburg, Sweden

**Keywords:** COPD, fibroblast, senescence, small airway disease

## Abstract

Small airway disease (SAD) is a key early-stage pathology of chronic obstructive pulmonary disease (COPD). COPD is associated with cellular senescence whereby cells undergo growth arrest and express the senescence-associated secretory phenotype (SASP) leading to chronic inflammation and tissue remodeling. Parenchymal-derived fibroblasts have been shown to display senescent properties in COPD, however small airway fibroblasts (SAFs) have not been investigated. Therefore, this study investigated the role of these cells in COPD and their potential contribution to SAD. To investigate the senescent and fibrotic phenotype of SAF in COPD, SAFs were isolated from nonsmoker, smoker, and COPD lung resection tissue (*n* = 9–17 donors). Senescence and fibrotic marker expressions were determined using iCELLigence (proliferation), qPCR, Seahorse assay, and ELISAs. COPD SAFs were further enriched for senescent cells using FACSAria Fusion based on cell size and autofluorescence (10% largest/autofluorescent vs. 10% smallest/nonautofluorescent). The phenotype of the senescence-enriched population was investigated using RNA sequencing and pathway analysis. Markers of senescence were observed in COPD SAFs, including senescence-associated β-galactosidase, SASP release, and reduced proliferation. Because the pathways driving this phenotype were unclear, we used cell sorting to enrich senescent COPD SAFs. This population displayed increased p21^CIP1^ and p16^INK4a^ expression and mitochondrial dysfunction. RNA sequencing suggested these senescent cells express genes involved in oxidative stress response, fibrosis, and mitochondrial dysfunction pathways. These data suggest COPD SAFs are senescent and may be associated with fibrotic properties and mitochondrial dysfunction. Further understanding of cellular senescence in SAFs may lead to potential therapies to limit SAD progression.

**NEW & NOTEWORTHY** Fibroblasts and senescence are thought to play key roles in the pathogenesis of small airway disease and COPD; however, the characteristics of small airway-derived fibroblasts are not well explored. In this study we isolate and enrich the senescent small airway-derived fibroblast (SAF) population from COPD lungs and explore the pathways driving this phenotype using bulk RNA-seq.

## Graphical abstract

**Figure:**
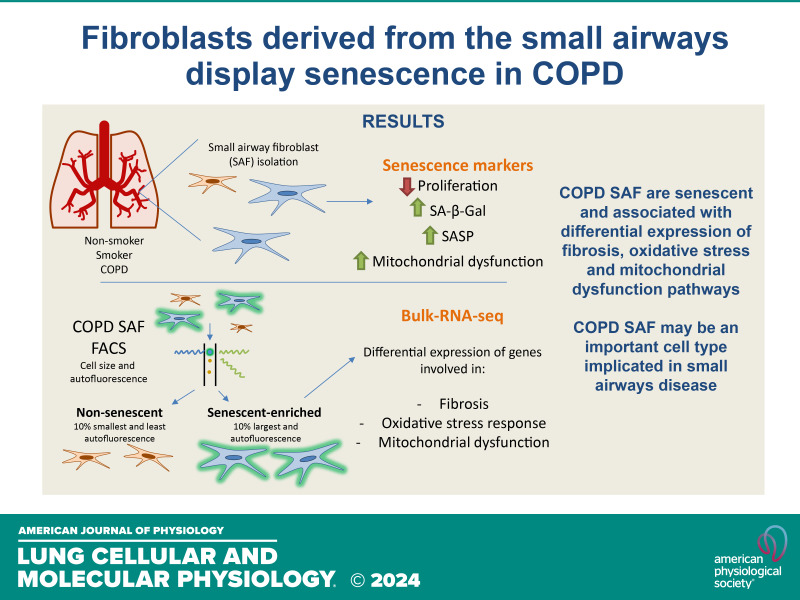

## INTRODUCTION

Chronic obstructive pulmonary disease (COPD) is an irreversible lung condition primarily involving three major pathologies: chronic bronchitis, small airway disease (SAD), and emphysema. Cigarette smoking is the major risk factor for COPD but exposure to other irritants such as air pollution and increasing age are also prominent risk factors (1). COPD is one of the top causes of death worldwide and the burden of this disease is predicted to increase in the coming years due to continued exposure to irritants and increasing age (2).

COPD is considered a disease of accelerated aging, with cellular senescence playing a key role in disease pathogenesis. Cellular senescence is defined as proliferative arrest of cells in response to various stressors, such as reactive oxygen species (ROS) and oxidative stress derived from cigarette smoke. Senescent cells display various markers including mitochondrial dysfunction, expression of senescence-associated β-galactosidase (SA-β-Gal), and cycle inhibitor expression (such as p15^INK4b^, p16^INK4a^ and p21^CIP1^), as well as release of the senescence-associated secretory phenotype (SASP). Senescent cells also undergo morphological changes, including increased cell size and autofluorescent properties due to the accumulation of age-related pigment, lipofuscin (3). Many of these senescence markers are also observed in COPD, with similar inflammatory profiles observed in COPD and aging, which is characteristic of the SASP (3, 4). In addition, there is evidence for increased senescence of several cell types in COPD, including leukocytes, endothelial, pulmonary artery smooth muscle, and epithelial cells (5–8).

Differential expression of tissue repair genes is observed in COPD parenchymal and airway tissues (9), which is associated with the deposition of extracellular matrix within these compartments in disease (10, 11). Fibroblasts are structural cells whose primary role is to maintain the lung and repair damage (12), however, different phenotypes of fibroblasts are thought to reside within each lung compartment. Fibroblasts derived from airway and parenchymal tissues display altered fibrotic characteristics and responses to stimuli (13–16) and may contribute to opposing pathologies in COPD.

SAD is characterized by chronic inflammation and peribronchial fibrosis of the small airways and is challenging to identify using traditional lung function parameters. SAD has been suggested to precede severe emphysematous disease and proposed as an early marker of COPD progression (17). This is supported by the recent publication from the COPDGene epidemiological study, which suggested patients with airway-predominant disease more rapidly progress to advanced stages of COPD and was associated with elevated 5-year all-cause mortality (18). This highlights the importance of understanding the underlying pathogenesis of SAD.

Fibroblasts are thought to be key contributors to SAD pathology. Current literature assessing fibroblast senescence in COPD has focused on cells derived from the parenchyma (non-airway) or whole lung digest. These publications have demonstrated that COPD fibroblasts display altered function and senescent features compared with cells from healthy lung tissue (19–23). Furthermore, the literature suggests senescence may also be linked to a profibrotic phenotype (24, 25). However, the fibrotic and senescent features of small-airway fibroblasts (SAF) have not been studied previously.

The aim of this study was to characterize the senescence and fibrotic properties of fibroblasts derived from small-airway tissue. This study shows that COPD SAFs exhibit some features of senescence including SA-β-Gal staining, reduced proliferation, and altered mitochondrial function. However, due to the proliferative nature of nonsenescent cells, it can be difficult to explore fibroblast senescence. Therefore, we used fluorescent-activated cell sorting (FACS) to enrich for senescent COPD SAFs to better characterize the phenotype of these cells and explored potential pathways involved in these phenotypes using RNA sequencing. Some work presented in this paper has been published as an abstract (26, 27).

## METHODS

### Patients

COPD severity was graded according to the Global Initiative for COPD guidelines based on spirometry. Cells were extracted from lung tissues of patients undergoing lung resection surgery at the Royal Brompton Hospital. Tissue was obtained from subjects undergoing pulmonary resection surgery, transplant, or from tissue unsuitable for transplant. Subjects with COPD were predominantly GOLD Stage 3. All subjects gave written informed consent (study ethics number 15/SC/0101). Demographics can be found in Supplemental Table S1. Donors were matched across all groups for age. The smoker and COPD groups showed significantly higher smoking history but this was matched between the smoker and patients with COPD. Patients with COPD exhibited significantly reduced forced expiratory volume in one second (FEV_1_) and FEV_1_/forced vital capacity (FVC) ratio compared with the nonsmoker and smoker groups.

### Small Airway and Parenchymal Fibroblast Isolation

Fibroblasts were isolated using explant culture of airway and parenchymal tissues isolated from the same peripheral lung tissue. In brief, fresh human lung tissue was flushed using Hanks’ balanced salt solution (HBSS). Both visible small airway (<2 mm in diameter) and surrounding parenchymal tissues were isolated from the same peripheral lung tissue using forceps and scissors. Between 3–5 samples of 0.5 cm^2^ airway and parenchymal samples (from multiple areas of the lung) were transferred to T25 flasks; these were cultured for fibroblasts at 37°C, 5% (vol/vol) CO_2_ in 3 mL of DMEM (Sigma-Aldrich, UK), 10% (vol/vol) FBS (BioSera, UK), 1% (vol/vol) l-glutamine (Sigma-Aldrich, UK), 1% (vol/vol) penicillin/streptomycin, and 1% (vol/vol) amphotericin (Gibco, UK) (Complete DMEM). Explant cultures were maintained for 2 wk (with media changes every 4 days) to allow growth of fibroblasts. After this time, tissue was removed and cells were passaged. Fibroblasts showed typical fibroblast morphology and were positively stained for vimentin and were negative for pan-cytokeratin. Cells were used between passages 2 and 5.

### Cell Treatments

For the hydrogen peroxide (H_2_O_2_) model of senescence, cells were plated at the densities specified below and allowed to adhere overnight. Cells were serum starved (0% FBS) for 24 h and treated with 200–500 µM H_2_O_2_ (Sigma, UK) for 48 h before collection.

### SA-β-Galactosidase Staining

Cells were plated at 4 × 10^4^ cells/well in Nunc removable eight-well chamber slides for 48 h. SAFs were stained according to manufacturer’s instructions (Abcam, UK). For analysis, slides were stained with 5 µM SYTO-16 nucleic acid stain (Life Technologies, UK). Cells were imaged for SYTO 16 and SA-β-Gal staining at ×20 magnification on a Zeiss Axioplan microscope. Three images were taken per well, selected based on SYTO 16 staining to avoid bias. Total cell count was quantified using the SYTO16 stain and cell counter function on ImageJ, and the number of SA-β-Gal-positive cells was expressed as a percentage of the total cell count.

### Proliferation Assay

SAFs were seeded in eight-well iCELLigence E-plates at 4 × 10^4^ cells/well and loaded into an iCELLigence instrument (ACEA Biosciences, Inc.), data were recorded from the proliferative phase of growth. To assess cell cycle arrest, cells were fixed and permeabilized in 70% ethanol and suspended in 3 µM propidium iodide (PI) (Invitrogen, UK). Staining was analyzed on a BD Accuri.

### qPCR

Primers were obtained from Life-technologies (Paisley, UK): *GNB2L1* (Hs00272002), *ACTA2* (Hs00426835), *CDKN1A* (p21^Cip1/Waf1^) (Hs00355782), *CDKN2A* (p16^INK4a^) (Hs00923894), *CDKN2B* (p15^INK4b^) (Hs00793225), *COL1A1* (Hs00164004), *COL3A1* (Hs00943809), *CXCL8* (Hs00174103), *DNM1L* (DRP-1) (Hs01552605), *FN-1* (Hs01549976), *IL-6* (Hs00174131) *MFN-1* (Hs00966851) *MFN-2* (Hs00208382), *SIRT1* (Hs01009006), and *TGFβ* primer (Hs00998133).

Cells were seeded at 7.5 × 10^4^ cells/well of a 24-well plate for 48 h and RNA was isolated from cells using miRNeasy mini kit (Qiagen Inc.) and reverse-transcribed using the high-capacity cDNA reverse transcription kit (Life Technologies, UK). Quantification of mRNA expression was determined by real-time quantitative PCR using TaqMan gene expression master mix (Life Technologies, UK). Guanine nucleotide-binding protein β polypeptide 2-like 1 (GNB2L1) was used as the housekeeping gene, and gene expression was quantified using the ΔΔCt method to determine relative fold change.

### Western Blotting

Antibodies used were p16^INK4a^ rabbit antibody (ab108349, 1:1,000, Abcam, UK), p21^Cip1/Waf1^ mouse antibody (DCS60) (CST2946, 1:1,000, Cell Signalling Technology, The Netherlands), and β-actin mouse antibody (ab6276, 1:50,000, Abcam, UK).

Cells were seeded at 3 × 10^5^ cells/well of a six-well plate and cultured for 48 h. Protein extracts were prepared using RIPA buffer (Sigma-Aldrich, UK) with protease inhibitors (Roche, UK). Extracts were analyzed by SDS-PAGE (Invitrogen, UK) and detected with Western blot analysis by chemiluminescence using an Odyssey Fc Imaging System. Band intensities were quantified by densitometry using the Li-Cor Image Studio Software. Full blot images can be found in the online supplement (https://doi.org/10.6084/m9.figshare.24175494).

### SASP Measurements

Supernatants were collected from protein expression experiments and SASP markers, plasminogen activator inhibitor-1 (PAI-1), interleukin(IL)-6, and C-X-C Motif Chemokine Ligand 8 (CXCL8) were measured in culture supernatants using enzyme-linked immunosorbent assays (Life Technologies, Paisley, UK and R&D Systems, Abingdon, UK). The lower limit of detection for the ELISAs were: IL-6: 0.021 ng/mL, CXCL8: 0.4 μg/mL, and PAI-1: 0.14 ng/mL. For zymography, supernatants were analyzed using SDS-PAGE on a 10% (wt/vol) gelatin gel and matrix metalloproteinase (MMP) activity visualized with colloidal blue stain (Invitrogen, Paisley, UK).

### Mitochondrial Function

Oxygen consumption rate (OCR) and extracellular acidification rate (ECAR) were measured using a Seahorse XF analyzer as a measure of mitochondrial respiration and glycolysis, respectively. Cells were plated onto eight-well Seahorse miniplates at 2 × 10^4^ cells/well. Cell Mito stress test and ATP rate assay were performed in Seahorse DMEM according to manufacturer instructions (Agilent, Cheshire, UK). Data was normalized to protein content as determined by a Bradford assay. To assess mitochondrial ROS production, cells were stained with 5 µM MITOSOX according to manufacturer’s instructions (Invitrogen, UK).

### Collagen Immunofluorescence

Cells were plated at 4 × 10^4^ cells/well on eight-well chamber slides. At 80% confluency, cells were fixed in 4% (vol/vol) paraformaldehyde (PFA). Cells were permeabilized and blocked in 0.5% (vol/vol) Triton X-100 and 5% (vol/vol) BSA. Slides were incubated overnight in either anti-collagen I (1:500, ab34710), anti-collagen III (1:200, ab7778), or rabbit IgG isotype control (1:200, ab171870) (Abcam, Cambridge, UK). Subsequently, they were incubated in Alexa Fluor 647-conjugated anti-rabbit antibody (1:200, ab150075, Abcam) for 3 h. Cells were counterstained with DAPI. Slides were imaged on an Inverted Widefield Microscope using Zen software. Three images were taken per well, selected using the DAPI staining to avoid bias. Fluorescence intensity of collagen staining was measured using ImageJ. A background fluorescence was also measured and subtracted from collagen-stained images to normalize between donors. Cell count was calculated using the DAPI stain and cell counter function.

### Enrichment of Senescent Fibroblasts

Senescent fibroblasts were enriched using a FACS methodology detailed by Hewitt et al. (28). In brief, SAFs were gated for 10% largest and most autofluorescent (FSC^high^FITC^high^) and 10% smallest and least autofluorescence (FSC^low^FITC^low^) based on forward scatter and FITC channels. Cells were sorted using a FACS Aria Fusion. Post-sorting, sorting efficiency was checked by recording 5,000 events. Post-sorting cells were counted and either plated as detailed above for immunofluorescence, SA-β-Gal staining, Seahorse, or proliferation overnight. For RNA and protein, the output of sorting a T125 flask of confluent cells was pelleted and stored at −80°C until processed.

### RNA Sequencing

Six COPD SAF samples that were enriched using this technique (samples shown in Figs. 4, 5, and 6) were submitted for RNA sequencing to explore novel pathways modulated by senescence. RNA sequencing was performed by Source Bioscience (www.sourcebioscience.com). Libraries were generated using TruSeq Stranded mRNA kit (Illumina). Libraries QC was performed by Agilent Bioanalyzer. Sequencing was performed using two lanes of the HiSeq 4000 for 75-bp paired-end reads.

Fastq were processed via the bcbio-nextgen v1.1.6 pipeline with Hisat2 (v2.1.0) as the aligner and Salmon (v0.14.1) as the quasi-mapper. QC was assessed using MultiQC (29). The reference genome was hg38, ensembl annotation release 94. For downstream analysis, the transcripts per million (TPM) values generated from Salmon were used as the expression values. Differential expression was performed using DESeq2 (v1.24.0) to generate multiple testing adjusted *P* values. Data can be found in Supplemental Table S2. RNA used for qPCR validation was sent for RNA-seq.

Pathway enrichment of differentially expressed genes was generated through Ingenuity Pathway Analysis (IPA, QIAGEN Inc.). Data are displayed as IPA-generated –log(*P* value) and ratio of proteins present in each pathway. Other visualizations were performed using R software (http://www.Rproject.org), ggplot2.

### Statistical Analysis

All data are expressed as individual data points and/or means ± SE. Data were analyzed using GraphPad Prism software (San Diego, CA). Paired data, between two groups, was analyzed using a Wilcoxon matched pairs signed ranks test. Comparisons between two independent datasets were analyzed using a Mann–Whitney *U* test. Comparisons between three or more independent subject/treatment groups were analyzed using a Kruskal–Wallis test with Dunn’s post hoc analysis. Data from iCELLigenece were analyzed using a repeated-measures two-way ANOVA. *P* values were considered significant if less than 0.05, RNA sequencing *P*-values were adjusted for multiple testing.

## RESULTS

### Small Airway Fibroblasts Display Higher *p21^CIP1^* Expression

**Figure 1.**
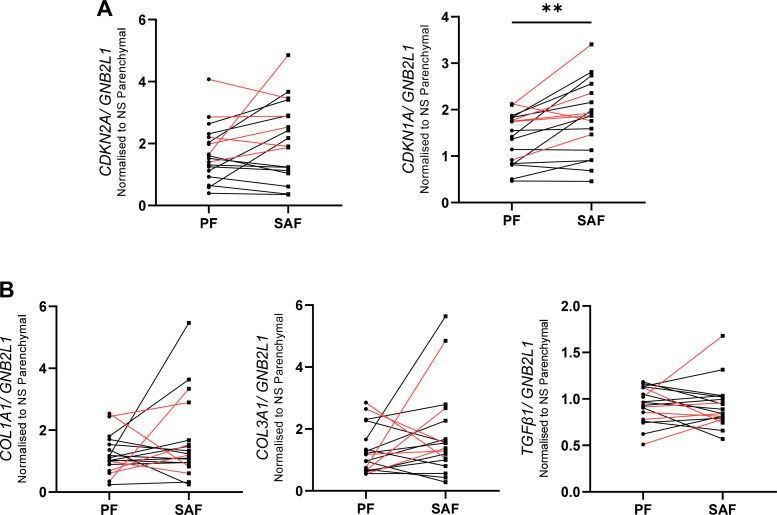
Expression of fibrotic and senescence markers in fibroblasts derived from small airway and parenchymal tissue from matched donors. Fibroblasts were grown from airway and parenchymal lung tissue of the same donors (*n* = 18 donors). Samples isolated from COPD lungs are shown in red and control in black. Gene expression of senescence markers *CDKN2A* (p16) and *CDKN1A* (p21) (*A*) and fibrotic markers *COL1A1, COL3A1,* and *TGFβ1* (*B*) was assessed using qPCR in parenchymal (●) and small airway fibroblasts (■). Data were analyzed using a Wilcoxon test: **P < 0.01. COPD, chronic obstructive pulmonary disease; PF, parenchymal fibroblast; SAF, small airway fibroblast.

We first explored the senescent and fibrotic characteristics of parenchymal and small airway-derived lung fibroblasts from matched donors. Gene expression of cell cycle inhibitor, *p21^CIP1^*, was significantly higher in SAF (*P* < 0.01), but there were no differences in *p16^INK4a^* expression (Fig. 1*A*). Of the fibrosis genes assessed, there was no difference in the expression of *TGFβ1*, *COL1A1*, or *COL3A1* (Fig. 1*B*).

### COPD SAFs Display an Increase in Some Senescence Markers

**Figure 2.**
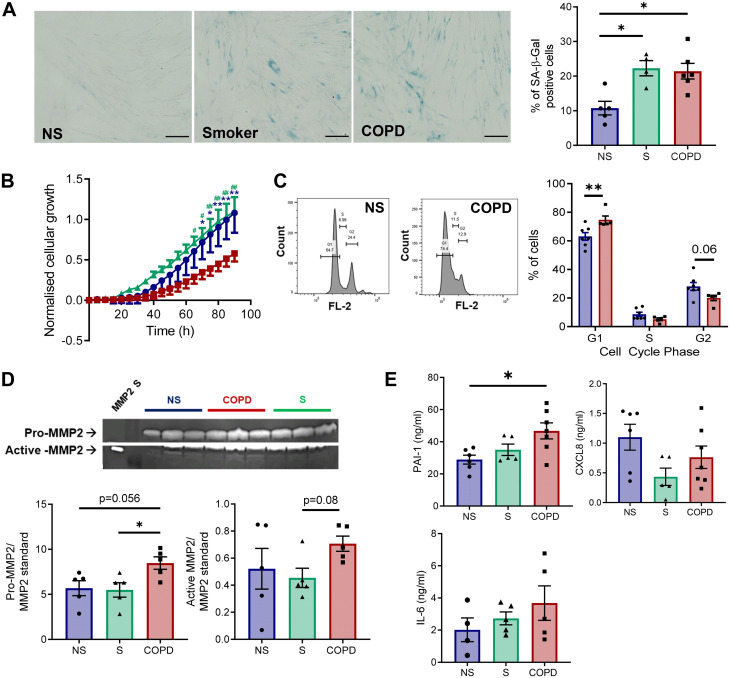
Cellular senescence markers in COPD SAFs. *A*: representative images of SA-β-Gal staining and quantification for percent positive SAF for SA-β-Gal NS (●), S (▴), and COPD (■) SAFs (*n* = 4–6 donors). *B*: proliferation rate of NS, S, and COPD SAFs as measured by iCELLigence technology (*n* = 4–7 donors). *C*: representative flow cytometry plots of propidium iodide staining and quantification for stage of cell cycle arrest (*n* = 5–7 donors). *D*: representative image of zymography gelatin gel and quantification for pro and active-MMP2 (*n* = 5 donors). *E*: release of SASP markers PAI-1, IL-6, and CXCL8 in SAF (*n* = 4–7 donors). Data are represented as individual data points and/or means ± SE. iCELLigence data were analyzed using a repeated-measures two-way ANOVA. Cell cycle data were analyzed using a Mann–Whitney *U* test. Comparisons between NS, S, and COPD groups are with a Kruskall–Wallis and Dunn’s post hoc analysis. *P < 0.05, **P < 0.01 compared with NS. #*P* < 0.05, ##*P* < 0.01 compared with S. Scale bars are 100 µm. COPD, chronic obstructive pulmonary disease; MMP2 S, matrix metalloproteinase-2 standard; NS, nonsmoker; S, smoker; SAF, small airway fibroblast; SA-β-Gal, senescence-associated β-galactosidase; SASP, senescence-associated secretory phenotype.

We next explored the expression of senescence markers at baseline in SAFs from nonsmokers, smokers, and patients with COPD. COPD and smoker SAFs displayed senescence markers including increased SA-β-Gal compared with nonsmoker SAFs (*P* < 0.05, Fig. 2*A*). COPD SAFs also had a reduced proliferation rate compared with nonsmoker and smoker SAFs (*P* < 0.01, Fig. 2*B*), and PI staining suggested that COPD SAFs were arrested in the G1 phase of the cell cycle (*P* < 0.01, Fig. 2*C*). COPD SAFs also displayed increased release of some SASP, including MMP2 (*P* = 0.05, Fig. 2*D*) and PAI-1 (*P* < 0.05, Fig. 2*E*) compared with nonsmoker SAFs. A similar trend was observed for IL-6 release, but no change in CXCL8 (Fig. 2*E*). While general markers of senescence were elevated in COPD SAFs, the gene and/or protein expression of cell cycle inhibitors *p15^INK4B^*, p16^INK4a^, and p21^CIP1^ was not elevated in COPD SAFs compared with nonsmoker or smoker SAFs (Supplemental Fig. S1).

### Senescence-Enriched COPD SAFs Display Elevated Senescence

These data suggest an increase in some senescence markers, but no change in cell cycle inhibitor levels in COPD SAFs. As fibroblasts are highly proliferative, it is unlikely that all cells are senescent. Nonsenescent cells may maintain proliferative capacity and dilute the expression of senescence markers measured. Therefore, we used a FACS method to enrich for senescent cells (28).

**Figure 3.**
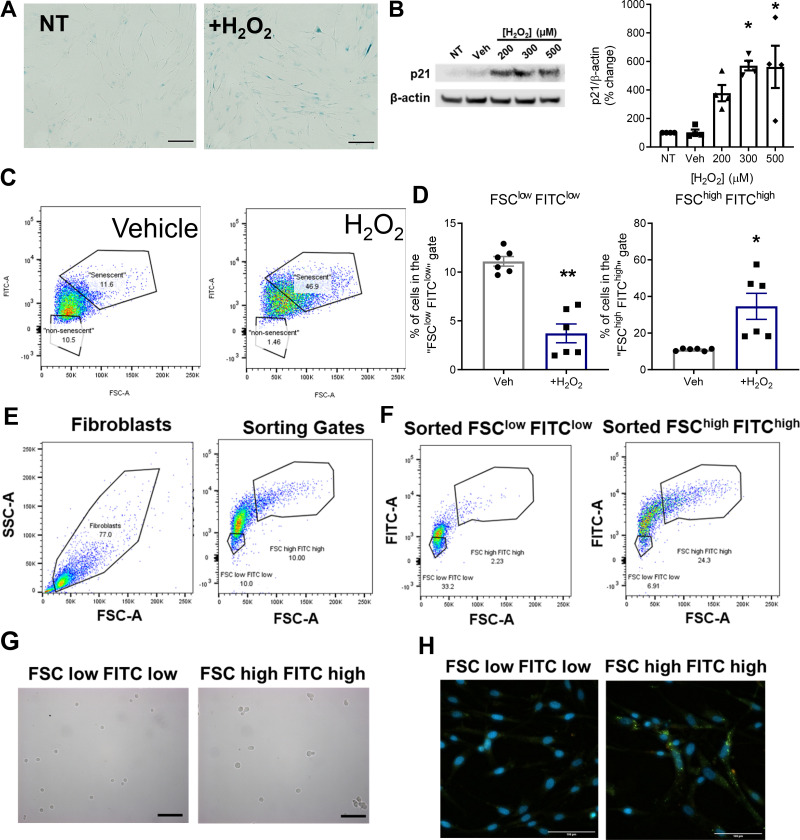
Change in fibroblast cell size and autofluorescence in senescence and gating strategy for enrichment via FACS. Fibroblasts from nonsmokers were treated with 500 µM H_2_O_2_ or untreated for 48 h. H_2_O_2_ induced expression of senescence markers. *A*: representative images of senescence-associated β-galactosidase staining. *B*: representative Western and quantification of p21 expression after H_2_O_2_ treatment (*n* = 4 donors). *C*: representative flow cytometry plots from vehicle (water) and H_2_O_2_-treated cells gated into “non-senescent” and “senescent” by their size and autofluorescence in the FITC channel. *D*: quantification of the change in the percentage of cells in the “non-senescent” and “senescent” gates with H_2_O_2_ treatment (72 h). *E*: sorting strategy to enrich for senescent COPD fibroblasts based on cell size and autofluorescence. Cells are gated to exclude debris and sorted into 10% smallest and least autofluorescence (FSC^low^FITC^low^) and 10% largest and most autofluorescence (FSC^high^FITC^high^). *F*: after sorting, cells were checked for sorting accuracy by recording 5,000 events. Representative images showing differences in cell size (*G*) and autofluorescence (*H*) post sort. Data are represented as individual data points and/or means ± SE. Western blot data were analyzed using a repeated-measures two-way ANOVA and changes in FACS populations were analyzed using a Wilcoxon test. *P < 0.05 and **P < 0.01 compared with vehicle. Scale bars are 100 µm. COPD, chronic obstructive pulmonary disease; H_2_O_2_, hydrogen peroxide; SAF, small airway fibroblast.

Hydrogen peroxide can be used to model senescence and induces the expression of markers such as SA-β-Gal and p21^CIP1^ in nonsmoker SAFs (Fig. 3, *A* and *B*). Senescent cells are also characterized by increased cell size and autofluorescence; these changes were observed in this hydrogen peroxide-induced senescence model (Fig. 3, *C* and *D*). Using this, we enriched for senescent SAFs using cell size and autofluorescence to allow us to better understand SAF senescence characteristics in COPD (Fig. 3*E*). After sorting, samples were checked for sorting efficacy, cell size, and autofluorescence (Fig. 3, *F*–*H*).

**Figure 4.**
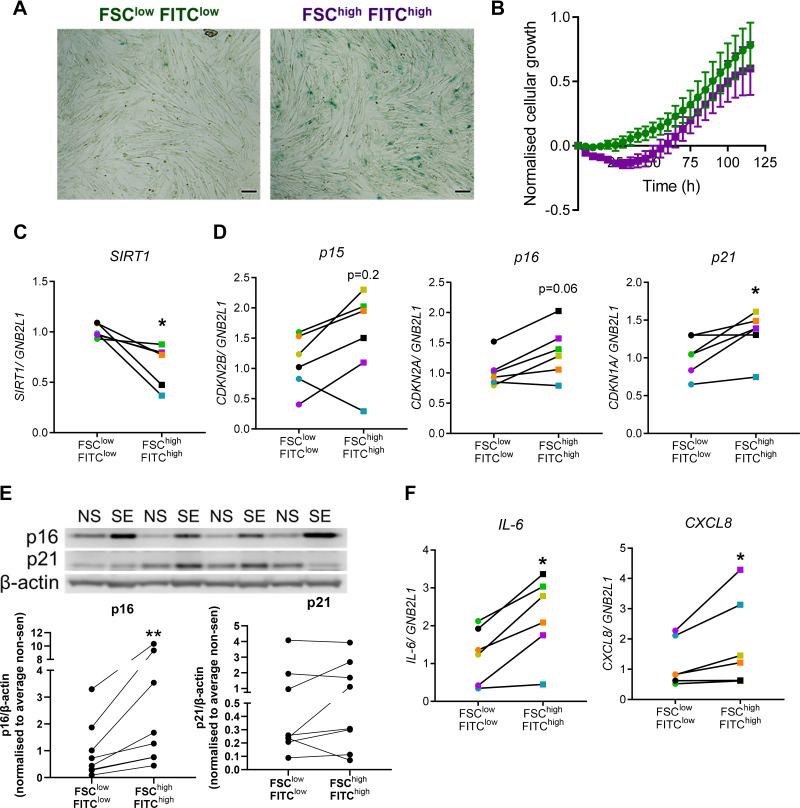
Expression of senescence markers in enriched senescent COPD SAF. SAF from COPD donors were sorted using FACS and sorted into 10% smallest and least autoflourescence (FSC^low^FITC^low^, ●) and 10% largest and most autofluorescence (FSC^high^FITC^high^, ■). Sorted populations were assessed for senescence markers. *A*: shows staining for senescence-associated-β-galactosidase. *B*: proliferation rate of sorted populations using iCELLigence. Samples were also collected for qPCR, where the expression of antiaging protein *SIRT1* (*C*) and cell cycle inhibitors (*D*) *CDKN2B* (p15), *CDKN2A* (p16), and *CDKN2A* (p21) was assessed (*n* = 6 donors). *E*: protein expression of p21^CIP1^ and p16^INK4a^ in sorted COPD SAF (*n* = 8 donors). *F*: gene expression of SASP markers *IL-6* and *CXCL8*. Data are represented as individual data points. Data were analyzed using a Wilcoxon test. *P < 0.05, **P < 0.01. Scale bars are 100 μm. COPD, chronic obstructive pulmonary disease; NS, nonsenescent (FSC^low^FITC^low^); SAF, small airway fibroblast; SE, senescence-enriched (FSC^high^FITC^high^).

We found that FSC^high^FITC^high^ COPD SAFs showed enrichment for SA-β-Gal-positive cells compared with FSC^low^FITC^low^ SAFs (Fig. 4*A*). These populations were cultured after sorting and FSC^high^FITC^high^ COPD SAFs displayed a lag in proliferation over the first 48 h compared with FSC^low^FITC^low^ SAFs (Fig. 4*B*). FSC^high^FITC^high^ SAFs displayed decreased expression of antiaging molecule, *SIRT1* (*P* < 0.05, Fig. 4*C*) and significantly higher *p21^CIP1^* expression (*P* < 0.05). Trends in higher expression of *p15^INK4B^* and *p16^INK4a^* compared with FSC^low^FITC^low^ SAFs were also observed (Fig. 4*D*). Protein expression of p16^INK4a^ was higher in FSC^high^FITC^high^ SAFs (*P* < 0.01), but p21^CIP1^ was not consistently increased (Fig. 4*E*). FSC^high^FITC^high^ SAFs also displayed increased expression and release of SASP markers *IL-6* and *CXCL8* (*P* < 0.05, Fig. 4*F* and Supplemental Fig. S2, *A–C*). These qPCR data were later confirmed in RNA-seq analysis (Supplemental Fig. S3, *A–C*).

SAFs from smokers were also sorted using the same strategy. These data also suggested FSC^high^FITC^high^ smoker SAFs potentially showing trends in increased expression of *p16*^INK4a^, *p21^CIP1^*, and *IL-6*. However, these cells did not display higher *CXCL8* expression (Supplemental Fig. S4). Overall, this suggests that FACS can be used to isolate a senescent-enriched population of primary lung fibroblasts, and that in COPD, these cells display markers of senescence including the expression of cell cycle inhibitors.

### Senescence-Enriched COPD SAFs Show Altered Mitochondrial Function

**Figure 5.**
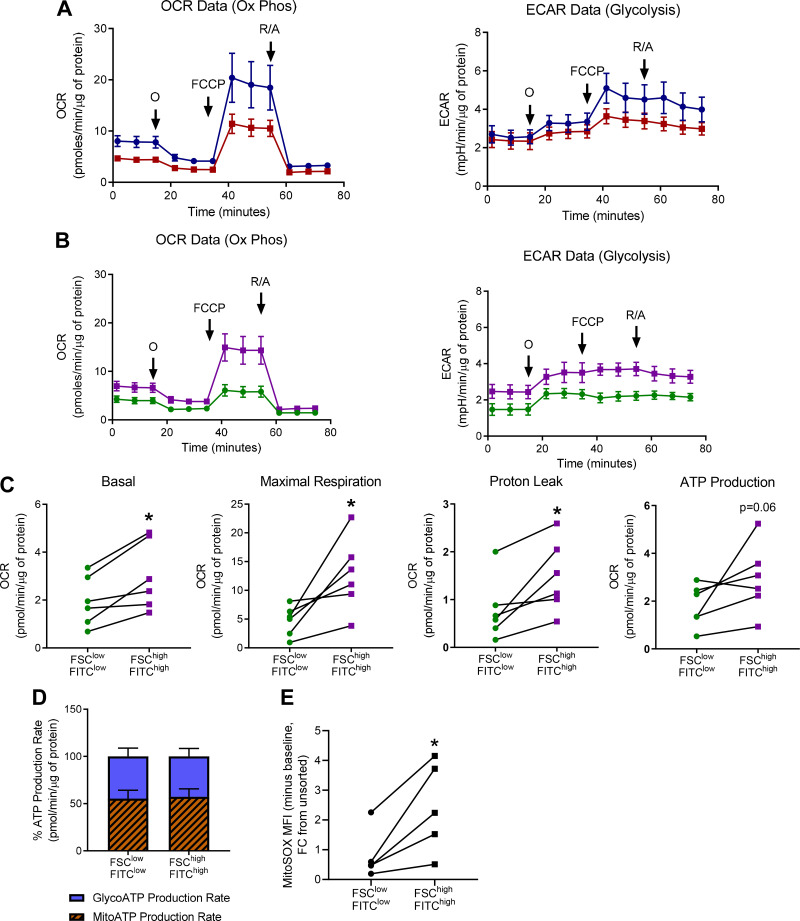
Mitochondrial function of senescent-enriched COPD SAFs. OCR (oxidative phosphorylation measure) and ECAR (glycolysis) traces from Cell Mito stress test in NS (●) and COPD (■) SAF (*A*) and FSC^low^FITC^low^ (●) and FSC^high^FITC^high^ (■) (*B*) sorted COPD SAFs. *C*: measurements of mitochondrial function parameters: basal respiration, maximal respiration, proton leak and ATP production in sorted COPD SAF populations. *D*: plot from ATP rate Seahorse assay showing distribution of ATP from glycolysis and oxidative phosphorylation (*n* = 6 donors) in sorted COPD SAF populations. *E*: mitochondrial ROS production in sorted COPD SAF populations (*n* = 5 donors). Data are represented as individual data points and/or means ± SE. Data are analyzed using a Wilcoxon test or a Friedman test with Dunn’s post hoc analysis. *P < 0.05. ATP, adenosine triphosphate; COPD, chronic obstructive pulmonary disease; ECAR, extracellular acidification rate; FCCP, carbonyl cyanide-*p*-trifluoromethoxyphenylhydrazone; O, oligomycin; OCR, oxygen consumption rate; R/A, rotenone and antimycin; SAF, small airway fibroblast.

In disease, mitochondria can become dysfunctional and lead to ROS production, this can negatively impact cellular function and promote senescence (30). Therefore, we explored whether this was also true in senescent COPD SAFs. At baseline, COPD SAFs displayed decreased mitochondrial capacity (Fig. 5*A* and Supplemental Fig. S5, *A–D*); however, FSC^high^FITC^high^ SAFs displayed increased mitochondrial function compared with nonsenescent COPD SAFs (Fig. 5*B*). Senescence-enriched COPD SAFs displayed increased mitochondrial parameters such as basal respiration, maximal respiration, proton leak (*P* < 0.05), and a trend in increased ATP production compared with nonsenescent SAFs (*P* = 0.06, Fig. 5*C*). Others have suggested increased mitochondrial function in senescence occurs alongside a shift in ATP production through glycolysis (31), however, no difference in the distribution of ATP from glycolysis and oxidative phosphorylation was observed here (Fig. 5*D*). Although senescence-enriched COPD SAFs show increased mitochondrial function, they may still be dysfunctional. MITOSOX staining suggested that the FSC^high^FITC^high^ SAFs display increased mitochondrial ROS (*P* < 0.05, Fig. 5*E*), suggesting the mitochondria may be dysfunctional and could be contributing to the senescent phenotype of these cells. However, this altered mitochondrial activity and ROS production in the senescence-enriched population was not due to altered mitochondrial dynamics or biogenesis, but could be due to altered mitophagy as *PINK1* and *Parkin* were elevated in FSC^high^FITC^high^ SAFs (Supplemental Fig. S6, *A–C*).

### Senescence-Enriched COPD SAFs Display Altered Expression of Fibrotic Genes

**Figure 6.**
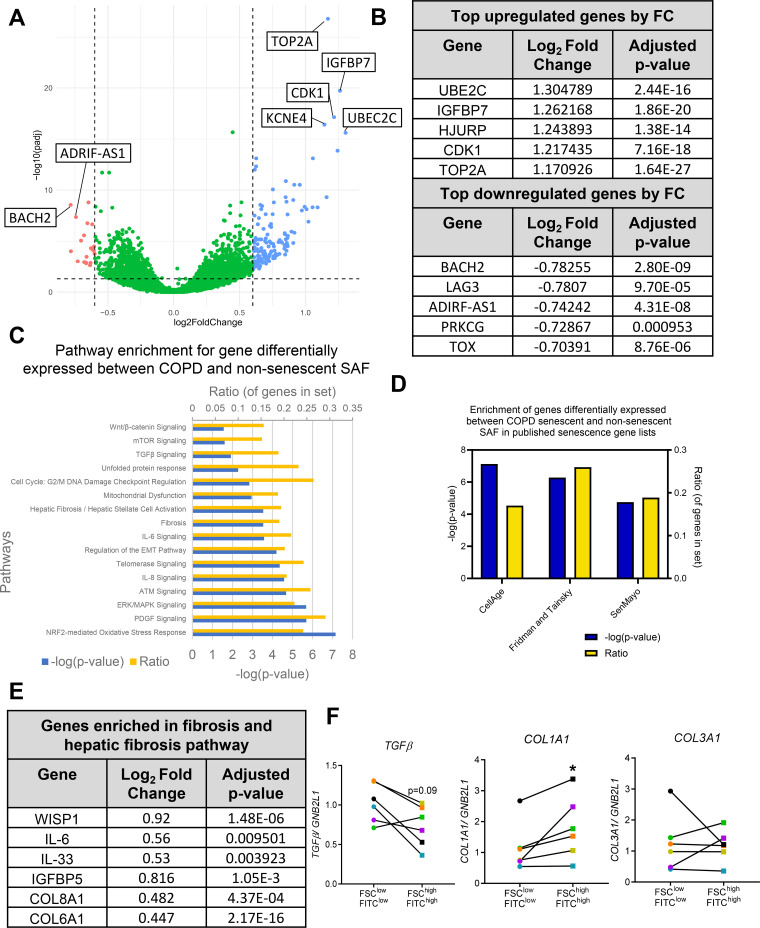
Altered pathways in senescence-enriched COPD SAF. Samples from FSC^low^FITC^low^ (●) and FSC^high^FITC^high^ (■) sorted COPD SAF were analyzed by RNA sequencing (*n* = 6 donors). *A*: volcano plot of differentially expressed genes in sorted senescence-enriched populations. Labeled are some of the top genes most upregulated and downregulated in senescence-enriched SAF. Horizontal dashed line shows *P*adj = 0.05. Vertical dashed lines show a fold change of 1.5 (*n* = 6 donors). *B*: summary of top 5 upregulated and downregulated genes based on Log2 fold change. *C*: ingenuity pathway analysis of significantly enriched canonical pathways based on differentially expressed genes from RNA-seq data (*n* = 6 donors). *D*: enrichment for previously published gene sets altered in senescence in this RNA dataset. *E*: differentially expressed genes involved in the fibrosis pathway enrichment. *F*: expression of fibrosis-related genes *TGFβ1*, *COL1A1*, and *COL3A1* in sorted COPD SAF (*n* = 6 donors). Data are represented as individual data points and/or mean. Data were analyzed using a Wilcoxon test. *P < 0.05. COPD, chronic obstructive pulmonary disease; SAF, small airway fibroblast.

As we have enriched for a senescence COPD SAF population, we next wanted to better understand what other pathways may be altered in these cells and associated with senescence and so RNA sequencing was performed (Supplemental Table S2). A volcano plot shows the distribution of differentially expressed genes in this dataset (Fig. 6*A*) and the table summarizes the top five up and downregulated genes (Fig. 6*B*). Ingenuity pathway analysis suggested that differentially expressed genes enriched for pathways associated with senescence and oxidative stress including NRF2 oxidative stress response, mitochondrial dysfunction, and mTOR signaling (Fig. 6*C*). This dataset also displayed enrichment of genes included in published senescence gene sets (32–34) (Fig. 6*D*). Full details of enriched pathways can be found in Supplemental Fig. S7.

Although COPD SAFs displayed no significant difference in fibrotic marker expression compared with nonsmoker or smoker SAFs (Supplemental Fig. S8), pathway analysis of the senescence-enriched COPD SAFs suggested enrichment for pathways associated with fibrosis, IL-6, TGFβ, and Wnt/β-catenin signaling (Fig. 6*C*). Some of the differentially expressed genes involved in promoting these pathways are shown in Fig. 6*E*. These included several genes that encoded collagen proteins (e.g., *COL1A2, COL6A1, COL6A3, COL8A1*), as well as mediators that have been suggested to promote fibrosis (e.g., *IL-6* and *IGFBP3-5*). Subsequently, we assessed the expression of fibrosis-related genes by qPCR. These data suggested FSC^high^FITC^high^ SAFs expressed lower levels of *TGFβ*, but higher levels of *COL1A1* (*P* < 0.05, Fig. 6*F*), however, only trends were observed after RNA-seq analysis (Supplemental Fig. S3*D*). Although there was no difference in the expression of other fibrosis markers including *COL3A1*, *fibronectin*, or alpha-smooth muscle actin *αSMA* (Supplemental Fig. S8*A*), immunocytochemistry staining of sorted SAFs suggested a trend in increased deposition of collagen 1, but not collagen 3 in FSC^high^FITC^high^ SAFs (Supplemental Fig. S9, *B* and *C*).

## DISCUSSION

Overall, our study demonstrates that SAFs from patients with COPD display markers of senescence. However, pathways driving this phenotype were unclear, therefore using FACS, we isolate a senescent-enriched population of COPD SAFs and demonstrate the senescence characteristics of these cells. RNA sequencing suggested increased expression of genes involved in fibrosis, mitochondrial dysfunction, and the oxidative stress response suggesting these may be associated with COPD SAF senescence.

Fibroblasts derived from the parenchyma have been shown to display increased markers of senescence in COPD (19–23). Bronchial biopsy airway fibroblasts also display some characteristics that can be attributed to senescence, such as larger size and less proliferative than those from the parenchyma (15, 35), but senescence hallmarks have not been fully explored in COPD SAFs. In this study, we observed increased expression of *p21^Cip1^* in SAFs compared with parenchymal-derived cells. This could be attributed to their location, as airway fibroblasts are closer to potential stimuli and injury (i.e., cigarette smoke exposure or epithelial damage). This suggests airway fibroblasts may also show senescence characteristics, but further experiments investigating protein and matrix deposition are needed to determine if these cells functionally differ.

Our data in SAFs are concordant with previous publications which have demonstrated increased SA-β-Gal (23, 36, 37), reduced proliferation (23, 38, 39), and SASP expression (20, 23) in COPD fibroblasts from lung digest or parenchymal tissue. However, unlike parenchymal COPD fibroblasts (23), COPD SAFs did not display increased release of SASP marker CXCL8, suggesting SASP may differ between fibroblast phenotypes. We also assessed other SASP mediators not previously explored in COPD fibroblasts. PAI-1 is a protease inhibitor which is an important regulator of fibrinolysis and has roles in fibrosis (40). Data presented here suggest that PAI-1 is elevated in COPD SAFs and may contribute to the elevated levels observed in COPD and could be a mediator of small-airway fibrosis (41, 42). This senescent phenotype observed in the COPD airway fibroblasts could contribute toward SAD by promoting local airway fibrosis and by potentially reduced ability to repair the lung appropriately; however, further work is required to link this cell phenotype to in situ disease pathology. In addition, not all senescence markers have been explored here, and while shortened telomeres is not observed in COPD parenchymal fibroblasts (22), telomere length, SA-β-Gal, and senescence-associated heterochromatin foci could also be assessed to further support the senescent phenotype.

Although COPD SAFs displayed some key markers of senescence, elevated expression of classical senescence pathways, such as p16^INK4a^ and p21^CIP1^, was not observed. SA-β-Gal staining suggested only ∼20% of cells were senescent, the remaining cells are likely to be nonsenescent cells which can continue to proliferate. These may dilute down the expression of senescent parameters, such as cell cycle inhibitors. Therefore, we used FACS to enrich for senescent COPD SAFs based on senescent cells displaying increased cell size and autofluorescence (3). This enabled us to better characterize the pathways mediating senescence, as well as other phenotypes associated with COPD SAFs; areas currently unexplored in the literature. This senescent-enriched population of COPD SAFs displayed classical senescence hallmarks including, SA-β-Gal and the SASP, as also seen in senescent-sorted fibroblast cell lines (28, 43). These cells also had increased expression of *p15^INK4B^*, p16^INK4a^, and *p21^CIP1^*, suggesting these are implicated in maintaining the COPD SAF senescent phenotype. We also presented preliminary data using the same sorting strategy in SAFs from smokers. These cells showed some similar trends, with increased expression of cell cycle inhibitors. But also, some differences, such as a trend decrease in *CXCL8*. This could suggest that senescent cells from smokers and patients with COPD display a different phenotype. One benefit of using this FACS method is that differences observed between the enriched cell population are unlikely to be due to a smoking effect, as cells come from the same donor and are therefore exposed to the same smoking environment. However, further FACS studies using cells from smokers without COPD and nonsmokers are necessary to understand the differences of senescent fibroblasts in COPD and control lungs.

We also explored the mitochondrial function of SAFs and the senescence-enriched population of COPD SAFs. A trend in reduced mitochondrial function has been demonstrated in COPD parenchymal fibroblasts (44), concordant with what was observed here. However, senescence-enriched COPD SAFs displayed increased mitochondrial function. These opposing results could be facilitated by the SASP from senescent fibroblasts altering mitochondrial regulation or represent differences between fibroblast subtypes yet to be explored. Increased mitochondrial activity in senescence seems contradictory as it is associated with growth arrest. However, this has been observed in senescent fibroblasts previously (31, 45, 46). Mitochondria are significant contributors to SASP development, but less important in proliferative arrest (30), and may relate to increased SASP observed here. We observed an increase in genes involved in mitophagy, this could suggest senescent cells recognize and are trying to remove dysfunctional mitochondria, but mitophagy may be impaired in some other way, however this requires further exploration and is beyond the scope of this study.

Increased mitochondrial mass has been demonstrated in senescent-enriched fibroblast cell lines (43). In addition, increased biogenesis is thought to be an adaptive response to oxidative stress, but can lead to senescence if prolonged (30). *SIRT1* expression was reduced in senescence-enriched COPD SAFs. Reduced SIRT1 is documented in COPD and is an antiaging molecule (7, 47, 48). SIRT1 can contribute to mitochondrial biogenesis through reduced activation of mTOR inhibitor, 5′ AMP-activated protein kinase (AMPK), as well as regulating the key mitochondrial controller PGC-1α. While we saw no differences in the expression of *PGC1α*, we have only explored gene expression here and would benefit from assessment of protein expression or electron microscopy to further understand changes in mitochondria.

RNA sequencing of the senescence-enriched COPD SAFs allowed us to explore other potential mechanisms that may be cause or consequence of this senescence phenotype. Differentially expressed genes were involved in the oxidative stress response, mTOR signaling, unfolded protein response, and mitochondrial dysfunction. Suggesting these areas are likely to be the contributing factors of this phenotype in COPD SAFs and require further exploration. Oxidative stress is thought to be a major contributor to the development of senescence and relevant to COPD. We observed increased mitochondrial ROS in COPD SAFs and the senescence-enriched population, and this has also been observed in sorted senescent-fibroblast cell lines (43). This suggests that ROS may be implicated in driving or at least sustaining the senescent phenotype of COPD SAFs. However, other mechanisms of senescence may also play a role. Epithelial cells, endothelial cells, and leukocytes also demonstrate senescence in COPD and produce SASP (5–8, 49). This can act on other cell types such as SAFs, and concordant with this, COPD fibroblasts are susceptible to senescence induction via SASP (23), which may also suggest some cell-cell communication. Replicative senescence can play a role in COPD parenchymal fibroblasts (23, 44); although shortened telomeres are observed in COPD (8), this has not been demonstrated in COPD parenchymal fibroblasts (22, 23), but could be a mechanism to explore in COPD SAFs.

Few differences were observed in fibrotic marker expression in COPD SAFs at baseline. It has been demonstrated that SMAD expression is similar between nonsmokers, smokers, and COPD parenchymal fibroblasts (21); however, this expression profile is altered in response to stimuli (e.g., TGFβ and cigarette smoke) and may explain why no differences were seen here between disease status. In addition, the profibrotic phenotype could be stimulated by the SASP, as opposed to directly associated with senescence, therefore, removal from the lung environment may also remove the profibrotic SASP from other cell types in the lung.

RNA sequencing did suggest association of senescent-enriched COPD SAFs with fibrotic characteristics. Cellular senescence is reported to be linked to pulmonary fibrosis (25, 50, 51) and the supernatant from senescent and idiopathic pulmonary fibrosis fibroblasts shown to have profibrotic effects (24, 29). In our RNA-seq dataset, differentially expressed genes were enriched in pathways involved in fibrosis, PDGF, TGFβ, and Wnt/β-catenin signaling. Preliminary experiments also suggested that collagen-I may be upregulated in senescent COPD SAFs and has previously been demonstrated as upregulated in COPD lungs (52). RNA sequencing also suggested upregulation of other profibrotic mediators and components in the senescence-enriched population (such as IL-33, IL-6, IGFBP5, and collagens). These have also been reported to be elevated in COPD and some also make up components of the SASP, suggesting SAF senescence could be a source of these profibrotic mediators and could be implicated in the pathogenesis of SAD.

Previous literature in parenchymal or lung digest-derived fibroblasts suggest that COPD cells display increased senescence markers compared with smoker cells (20, 22, 23). In this paper, many markers were also elevated in smoker SAFs. Traditional lung function parameters used to classify donors as COPD are measures of large airway obstruction. Therefore, it cannot be ruled out that some smokers could be experiencing mild disease or SAD which is not detected by traditional lung function parameters. In addition, senescence markers can be induced by cigarette smoke (53–55), so smokers may also display senescence but have other mechanisms that prevent deleterious effects. Many fibrotic markers were elevated in smoker SAFs as well, relative to nonsmoker and COPD SAFs. Extracellular matrix expression is thought be elevated in smokers/early disease (potentially those with SAD) but decline with COPD severity (9, 10). COPD donors in this study were mostly GOLD stage 3 and may be why few differences were observed. Inclusion of patients with milder COPD would be beneficial to assess if fibrotic and/or senescence characteristics are changed with disease progression in small airways.

A limitation of this study is methodology of SAF isolation. While fibroblasts were cultured from tissue derived from the small airways, the presence of other subtypes cannot be excluded. In addition, there are also limitations in isolation of fibroblasts from airways. First, not all COPD airways demonstrate SAD. Therefore, fibroblasts could have been grown from relatively healthy airways in a COPD lung. Second, the outgrowth method of fibroblast isolation relies on proliferation of cells. This technique may not be optimal for the isolation of in vivo senescent cells as they are less proliferative and may promote senescence of remaining cells.

Finally, it should also be considered that the nonsenescent COPD SAFs are also cells which are present in the lung and may play a role in COPD. These could represent an alternative phenotype of fibroblast. Further characterization of the fibroblast phenotypes that exist in the lung and COPD, especially airway and parenchymal fibroblasts, is needed to fully understand their contribution to COPD.

In conclusion, cellular senescence is a mechanism associated with aging and observed in accelerated aging diseases, such as COPD (3, 4). For the first time, this study explored the senescent and fibrotic phenotype of SAFs and demonstrated that COPD SAFs display increased markers of senescence. Using FACS, a senescence-enriched population of COPD SAFs was isolated, and RNA-sequencing data suggest the senescent phenotype may link to mitochondrial dysfunction and some fibrotic processes. Overall, SAFs may be distinct from parenchymal fibroblasts and understanding the phenotype of these cells may allow the development of targeted therapy against SAD.

## DATA AVAILABILITY

Data underlying the findings described in this manuscript may be obtained in accordance with AstraZeneca’s data sharing policy described at https://astrazenecagrouptrials.pharmacm.com/ST/Submission/Disclosure. Use the “Enquiries about Vivli Member Studies” (https://vivli.org/members/enquiries-about-studies-not-listed-on-the-vivli-platform/) form and include the publication title and data Accession number GSF1490425 in your request.

## SUPPLEMENTAL DATA

10.6084/m9.figshare.24175494 Supplemental Western blot images: https://doi.org/10.6084/m9.figshare.24175494.

10.6084/m9.figshare.24179442 Supplemental Fig. S1: https://doi.org/10.6084/m9.figshare.24179442.

10.6084/m9.figshare.24179448 Supplemental Fig. S2: https://doi.org/10.6084/m9.figshare.24179448.

10.6084/m9.figshare.24179472 Supplemental Fig. S3: https://doi.org/10.6084/m9.figshare.24179472.

10.6084/m9.figshare.24179553 Supplemental Fig. S4: https://doi.org/10.6084/m9.figshare.24179553.

10.6084/m9.figshare.24179574 Supplemental Fig. S5: https://doi.org/10.6084/m9.figshare.24179574.

10.6084/m9.figshare.24179577 Supplemental Fig. S6: https://doi.org/10.6084/m9.figshare.24179577.

10.6084/m9.figshare.24179589 Supplemental Fig. S7: https://doi.org/10.6084/m9.figshare.24179589.

10.6084/m9.figshare.24179595 Supplemental Fig. S8: https://doi.org/10.6084/m9.figshare.24179595.

10.6084/m9.figshare.24179610 Supplemental Fig. S9: https://doi.org/10.6084/m9.figshare.24179610.

10.6084/m9.figshare.24179634 Supplemental Table S1: https://doi.org/10.6084/m9.figshare.24179634.

10.6084/m9.figshare.24188565 Supplemental Table S2: https://doi.org/10.6084/m9.figshare.24188565.

## GRANTS

This work was supported by Imperial College London (Wellcome Trust - 208340/Z/17/Z, 104931/Z/14/Z and UK Research and Innovation | BBSRC - BB/L015129/1) and AstraZeneca. This study was funded by a National Heart and Lung Institute (NHLI) Foundation Studentship and an equipment grant from the Wellcome Trust (grant 208340/Z/17/Z).

## DISCLOSURES

C.L.W., S.M., and L.M. are or were employed by AstraZeneca and some may have stock or stock options. P.J.B. is Advisor and on scientific advisory board for AstraZeneca, Boehringer Ingelheim, Teva Lectures on behalf of AstraZeneca, Boehringer-Ingelheim, Novartis, Teva. None of the other authors has any conflicts of interest, financial or otherwise, to disclose.

## AUTHOR CONTRIBUTIONS

C.L.W., J.R.B., L.M., L.E.D., and P.J.B. conceived and designed research; C.L.W. and P.S.F. performed experiments; C.L.W. and S.M. analyzed data; C.L.W., J.R.B., S.M., and L.E.D. interpreted results of experiments; C.L.W. and S.M. prepared figures; C.L.W. drafted manuscript; C.L.W., J.R.B., S.M., L.M., L.E.D., and P.J.B. edited and revised manuscript; C.L.W., J.R.B., S.M., P.S.F., L.M., L.E.D., and P.J.B. approved final version of manuscript.
